# Dynamic interplay between CXCL levels in chronic Hepatitis C patients treated by Interferon

**DOI:** 10.1186/1743-422X-10-218

**Published:** 2013-07-01

**Authors:** Abdel-Rahman N Zekri, Abeer A Bahnassy, Waleed S Mohamed, Hanaa M Alam EL-Din, Hend I Shousha, Naglaa Zayed, Dina H Eldahshan, Ashraf Omar Abdel-Aziz

**Affiliations:** 1 Virology and Immunology Unit, Cancer Biology Department, National Cancer Institute, Cairo University, 1st Kasr El-Aini st, Cairo, Egypt; 2 Tropical Medicine Department, Faculty of Medicine, Cairo University, Kasr El-Aini st, Cairo, Egypt; 3 Pathology Department, National Cancer Institute, Cairo University, 1st Kasr El-Aini st, Cairo, Egypt; 4 Clinical Pathology Department, Faculty of Medicine, Benisuef University, Cairo, Egypt

**Keywords:** HCV, IFN, Chemokine’s, E-Cadherin

## Abstract

**Background:**

Combined pegylated interferon-α and ribavirin therapy has sustained virological response (SVR) rates of 54% to 61%. Pretreatment predictors of SVR to interferon therapy have not been fully investigated yet. The current study assesses a group of chemokines that may predict treatment response in Egyptian patients with chronic HCV infection.

**Patients and methods:**

CXCL5, CXCL9, CXCL11, CXCL12, CXCL 13, CXCL 16 chemokines and E-Cadherin were assayed in 57 chronic HCV patients’ sera using quantitative ELISA plate method. All studied patients were scheduled for combined pegylated interferon alpha and ribavirin therapy (32 patients received pegylated interferon α 2b, and 25 patients received pegylated interferon α 2a). Quantitative hepatitis C virus RNA was done by real time RT-PCR and HCV genotyping by INNOLIPAII.

**Results:**

There was no significant difference (p > 0.05) in baseline HCV RNA levels between responders and non-responders to interferon. A statistically significant difference in CXCL13 (p = 0.017) and E-Cadherin levels (P = 0.041) was reported between responders and nonresponders at week 12. Significant correlations were found between changes in the CXCL13 levels and CXCL9, CXCL16, E-cadherin levels as well as between changes in E-cadherin levels and both CXCL16 and ALT levels that were maintained during follow up. Also, significant changes have been found in the serum levels of CXCL5, CXCL13, and CXCL16 with time (before pegylated interferon α 2 a and α 2 b therapy, and at weeks 12 and 24) with no significant difference in relation to interferon type and response to treatment.

**Conclusion:**

Serum levels of CXCL13 and E-Cadherin could be used as surrogate markers to predict response of combined PEG IFN-α/RBV therapy, especially at week 12. However, an extended study including larger number of patients is needed for validation of these findings.

**Clinical trial No:**

NCT01758939

## Background

Hepatitis C virus (HCV) is a major cause of a wide spectrum of liver diseases ranging from mild chronic hepatitis to end stage cirrhosis and hepatocellular carcinoma. The World Health Organization (WHO) estimates that 170 million individuals worldwide are infected with HCV [1], However, the prevalence of HCV infection varies throughout the world. One decade ago, Frank et al., reported that Egypt had the highest number of reported HCV infections, largely attributed to the use of contaminated parenteral antischistosomal therapy [2]. The overall prevalence of HCV antibodies in the Egyptian population was 15% for age of 15 to 59 years and infection was higher among rural residents [3], and about 91% of the patients were infected with HCV genotype 4 [4].

Combined pegylated interferon (IFN)-α and ribavirin (RBV) therapy has sustained virological response (SVR) rates reaching up to 61% in different clinical trials for HCV Genotype 1 patients [5,6]. The therapeutic efficacy of PEG IFN-α/RBV is likely due to their antiviral and immune-modulatory properties [7]. Factors associated with SVR to interferon treatment have not been fully investigated in HCV genotype 4 infected patients. Previous studies demonstrated that age, body weight, baseline ALT, baseline HCV RNA viral load, HCV genotype and the level of fibrosis or cirrhosis are some predictors of response [8-12]. However, recent studies indicated that cytokines could be used as markers for disease progression in HCV infected patients [13].

Some recent studies demonstrate that IL-8, and CXCL10 chemokines correlate with poor response to antiviral therapy in CHC patients [13-15] or have prognostic utility as a marker of treatment outcome [16-18]. Moreover, control of HCV infection may depend in part on chemokine-mediated recruitment of specific T cells to the liver [19]. In this context, Moura et al., [17] evaluated the association between pretreatment plasma levels of chemokines CCL2, CCL3, CCL11, CCL24, CXCL9, CXCL10 and soluble tumor necrosis factor receptors and the virological response in CHC-treated patients. They found that pretreatment CXCL10 level predicts EVR and SVR to IFN-α and ribavirin and may therefore be useful in the evaluation of candidates for therapy.

However, no previous studies have been done in Egypt yet to assess the possible role of chemokines levels as predictors of response to interferon therapy. The current study aims to assess 1) the changes in serum levels of CXCL5, CXCL9, CXCL11, CXCL12, CXCL 13, CXCL 16 chemokines and E-Cadherin in Egyptian patients with chronic HCV genotype 4 infection who were recruited for combined PEG IFN-α/RBV therapy at baseline and after 12 and 24 weeks, and 2) to determine the utility of using these markers as predictors of the treatment response through determining the influence of therapy on these chemokines.

## Results

The current study was conducted on 57 CHC genotype-4 patient s, of which 32 (56.1%) received pegylated interferon α 2b, and 25 (30.4%) received pegylated interferon α 2a. Clinical and laboratory findings of the studied patients are shown in Table 1. There was no significant difference between pegintron and pegasys patients regarding histopathological and laboratory findings (p > 0.05) except for the age (p=0.04).

**Table 1:** Clinical and Laboratory findings of the studied 57 chronic hepatitis C patients

**Parameter**	**PEG-INTRON**	**PEGASYS**	**P-value**
**I. Demographic features**	N=32	N=25	
**-Age**	37.72 ± 8.44	42.28 ± 7.86	0.04
**-BMI (Kg/m2)**	26.35 ± 4.06	27.87 ± 4.13	0.17
**-Male**	27 (84.4%)	21 (84%)	0.96
**-Female**	5 (15.6%)	4 (16%)	
**-M:F ratio**	5.4:1	5.25:1	
**II. Pathological features**			
**-Necro inflamm.activity;**	**A1** : 13 (40.6%)	**A1** : 8 (32%)	0.52
	**A2 :** 10 (31.2%)	**A2 :** 10 (40%)	
	**A3** : 4 (12.5%)	**A3** : 5 (20%)	
	**A4** : 2 (6.2%)	**A4** : 1 (4%)	
**-Fibrosis**	**Missed**: 3 (9.3%)	**Missed**: 1 (4%)	
	**F1:** 17 (53.1%)	**F1:** 10 (40%)	0.87
	**F2 :** 4 (12.5%)	**F2 :** 4 (16%)	
	**F3 :** 4 (12.5%)	**F3 :** 3 (12%)	
	**F4** : 2 (6.2%)	**F4** : 2 (8%)	
**-Steatosis**	**Missed** : 5(15.6%)	**Missed** : 6 (24%)	
	**S0 :** 11 (34.3%)	**S0 :** 11 (44%)	0.53
	**S1:** 12 (37.5%)	**S1:** 12 (48%)	
	**S2:** 7 (21.8%)	**S2:** 7 (28%)	
	**S3:** 2 (6.2%)	**S3:** 2 (8%)	
**III. Laboratory features**	(Mean ± SD)		
**HCV PCR Log (EQ/ML)**	5.4431	5.3824	0.79
**(before treatment)**			
**WBC × 103/mm3**	5.9344 ± 1.87266	6.1600 ± 2.55261	0.70
**Hemoglobin (gm/dl)**	14.3 ± 1.5	12.1 ± 1.1	0.67
**Platelets count/mm3**	212148 ± 76965	150868.8 ± 1.0	0.32
**AST (0-42 IU/L)**	60.1 ± 38.5	63.7200 ± 41.67	0.21
**ALT (0-42 IU/L)**	72.6 ± 50.4	88.5 ± 48.6	0.31
**ALP (0-290 IU/L)**	117.7 ± 67.7	93.9 ± 72.6	0.14
**Total Bil(0.1-1.2 mg/dl)**	0.81 ± 0.41	.7689 ± .311	0.81
**Indirect Bil (mg/dl)**	0.45 ± 0.80	.4526 ± 29238	0.69
**Albumin (3.5-5.5gm/dl)**	4.21 ± 0.43	3.62 ± 0.32	0.34
**Prothrombin (%)**	77.0 ± 27.0	71.1± 12.5	0.60
**AFP (0-10 ng/ml)**	16.50 ± 9.76	10.1± 4.7	0.10

Baseline mean values of HCV RNA Log (EQ/ML) levels for the patients who received pegylated interferon α2b (Peg-intron) and pegylated interferon α 2a (Pegasys) were 5.4431 Log (EQ/ML) and 5.3824 Log (EQ/ML); respectively.

Different baseline data that might affect achievement of sustained virological response (SVR) such as age, body mass index, AST, ALT, and viral load were analyzed for pegylated interferon α2b and α2a therapy. There was a statistically significant difference between responders and non-responders in the AFP value for patients who received pegylated interferon α 2b (p=0.041) and α 2a (p = 0.05) therapy. Also, there was a statistically significant difference between responders and non-responders in AST value for patients who received pegylated interferon α2b (p=0.049) and α2a (0.001) therapy. On the other hand, the other baseline data did not differ significantly between the two studied groups (Table 2).

**Table 2:** Baseline differences of Lab findings among responders and non-responders of Pegylated interferon α 2b and α 2a therapy

**Parameter**	**Pegylated interferon α 2b**		**P-value**	**Pegylated interferon α 2a**		**P-value**
	**Non-responders (n=10)**	**Responders (n=22)**		**Non-responders (n=13)**	**Responders (n=12)**	
Age (Year)	38.70 ± 9.76	37.27 ± 7.98	0.66	43.69 ± 5.76	40.75 ± 9.37	0.35
BMI** (Kg/m2)	24.87 ± 4.63	27.02 ± 3.69	0.16	28.73 ± 3.99	26.93 ± 4.25	0.28
AST (0–42 IU/L)	39.20 ± 25.68	64.15 ± 30.54	0.049*	87.61 ± 43.13	37.83 ± 18.80	0.001*
ALT (0–42 IU/L)	44.12 ± 22.1	68.4 ± 21.1	0.07	46.42 ±14.13	43.56 ± 3.56	0.1
ALP (0–290 IU/L)	16.53 ± 9.7	4.66 ± 7.51	0.041*	13.94 ±15.38	4.24 ± 2.26	0.05*
HCVPCR Log (EQ/ML) before treatment	5.3082	5.4975	0.61	5.5034	5.1751	0.29

*P value <0.05 considered significant, BMI** : Body Mass Index.

**Figure 1:**
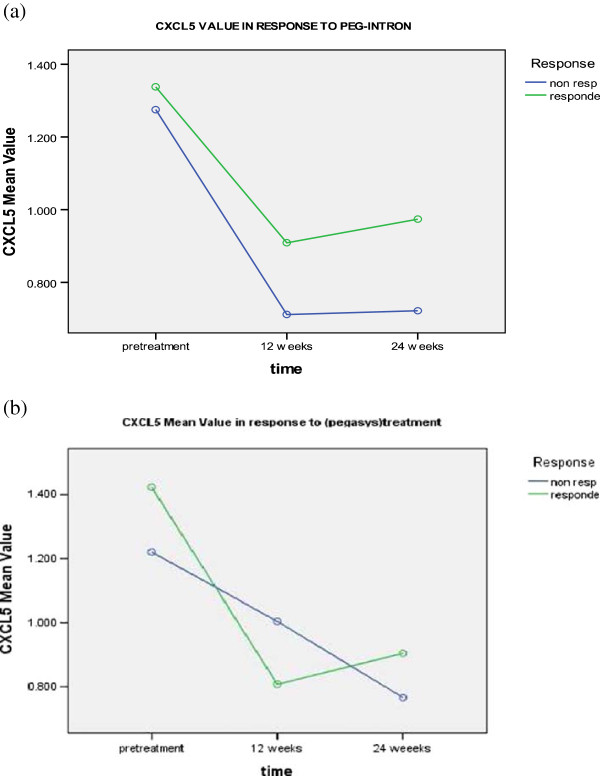
CXCL-5 levels in responders and non responders to a) PEG-Interferone and, b) pegasys at different time intervals.

Serum levels of the studied chemokines in response to pegylated interferon α2b and α2a therapy were determined before the beginning of treatment, after 12 and 24 weeks of treatment and correlated to the clinical data and response to treatment. However, full clinical and follow up data were available for 25 patients in group I (receiving Peg-intron) and 19 patients in group II (receiving pegasys treatment) only (Table 3). Each group was divided into two arms (responders and non-responders). As shown in Table 3 and Figure 1 the serum pretreatment levels of CXCL5 (1.275 ± 0.969 pg/ml) and (1.337 ± 0.792 pg/ml) were significantly higher than those assessed either at 12 (0.711 ± 0.459 pg/ml) and (0.908 ± 0.588 pg/ml) or 24 weeks (0.722 ± 0.466 pg/ml) and (0.951 ± .571 pg/ml) of pegylated interferon α2b therapy for both non-responders and responders; respectively.

**Table 3:** Different chemokine’s levels of 44 patients in response to treatment

**Parameter**		**Pegylated interferon α 2b (PEG-INTRON) (n=25)**		**Pegylated interferon α 2a (PEGASYS) (n=19)**		**P-value**
		**Non-responders (n=6)**	**Responders (n=19)**	**Non-responders (n=9)**	**Responders (n=10)**	
**CXCL5(pg/ml)**	**Pretreatment**	1.27500 ± .969563	1.33774 ± .792903	1.22044 ± 1.022862	1.42330 ± .925645	0.000^a^, 0.602^b^, 0.875^c^, 0.770^d^, 0.345^e^, 0.703^f^
	**12 Weeks**	.71167 ± .459045	.90895 ± .588673	1.00456 ± .699765	.80800 ± .609552	
	**24 Weeks**	.7220 ± .46685	.9518 ± .57106	.7664 ± .43326	.8625 ± .63422	
**CXCL9(pg/ml)**	**Pretreatment**	.19233 ± .114175	.20568 ± .076974	.18922 ± .074028	.18810 ± .063218	0.082^a^, 0.744^b^, 0.415^c,^ 0.962^d^, 0.199^e^, 0.517^f^
	**12 Weeks**	.19233 ± .124331	.14284 ± .040243	.16744 ± .127693	.12573 ± .053301	
	**24 Weeks**	.13750 ± .072116	.18945 ± .121548	.15544 ± .097306	.13464 ± .055773	
**CXCL11(pg/ml)**	**Pretreatment**	.13520 ± .080444	.13945 ± .072608	.17885 ± .093798	.15742 ± .100730	0.187^a^, 0.584^b^, 0.459^c^, 0.435^d^, 0.065^e^, 0.634^f^
	**12 Weeks**	.15260 ± .081185	.16190 ± .098519	.17154 ± .049891	.19991 ± .152004	
	**24 Weeks**	.14883 ± .038071	.17705 ± .102551	.12744 ± .044230	.19255 ± .161593	
**CXCL12(pg/ml)**	**Pretreatment**	84.69483 ± 206.396544	.39289 ± .154332	.55400 ± .244147	.45300 ± .163781	0.117^a^, 0.085^b^, 0.085^c^, 0.229^d^, 0.065^e^, 0.085^f^
	**12 Weeks**	.49400 ± .065023	.42263 ± .147961	.54833 ± .196068	.43490 ± .205439	
	**24 Weeks**	.373 ± .1015	.439 ± .1431	.496 ± .1217	.42900 ± .1590	
**CXCL13(pg/ml)**	**Pretreatment**	.34000 ± .069974	.31553 ± .105584	.40567 ± .274538	.29844 ± .105315	0.002^a^, 0.085^b^, 0.085^c^, 0.229^d^, 0.065^e^, 0.085^f^
	**12 Weeks**	.50483 ± .139194	.43342 ± .246432	.52944 ± .271026	.34133 ± .145816	
	**24 Weeks**	.47333 ± .181595	.53990 ± .377348	.62800 ± .455588	.39955 ± .128076	
**CXCL16(ng/ml)**	**Pretreatment**	.94633 ± .101567	.87458 ± .244527	1.14611 ± .424545	1.01250 ± .331579	0.000^a^, 0.221^b^, 0.527^c^, 0.689^d^, 0.577^e^, 0.866^f^
	**12 Weeks**	1.15333 ± .298719	1.11037 ± .323698	1.25411 ± .342009	1.24990 ± .417217	
	**24 Weeks**	1.26867 ± .353015	1.13953 ± .408803	1.29189 ± .354069	1.28820 ± .402766	
**E-cadherin(ng/ml)**	**Pretreatment**	.41983 **±** .425530	.40989 **±** .436687	.82833 **±** .843119	.68330 **±** .660015	0.140^a^, 0.274^b^, 0.749^c^, 0.504^d^, 0.578^e^, 0.794^f^
	**12 Weeks**	.72350 **±** .666040	.55905 **±** .577851	.92678 **±** .782186	.74020 **±** .657444	
	**24 Weeks**	.63783 **±** .634553	.77832 **±** .858560	.81578 ± .834449	.81032 **±** .779431	

*p-value comparing the effect of time*^a^*, treatment type*^b^*, response to treatment*^c^*, time-type interaction*^d^*, time-response interaction*^e^*, type-response interaction*^f^.

**Table 4:** Different chemokine’s levels of 55 patients at 12 weeks in response to treatment

**Parameter**		**Pegylated interferon α 2b (PEG-INTRON) (n=31)**		**Pegylated interferon α 2a (PEGASYS) (n=24)**		**P-value**
		**Non-responders (n=10)**	**Responders (n=21)**	**Non-responders (n=13)**	**Responders (n=11)**	
**CXCL5(pg/ml)**	**Pretreatment**	1.45250 **±** 940684	1.30990 **±** .759433	1.44500 **±** 1.077797	1.43018 **±** .878441	0.000^a^, 0.486^b^, 0.593^c^, 0.236^d^, 0.665^e^, 0.552^f^
	**12 Weeks**	.71740 **±** .377958	.88486 **±** .588673	1.25877 **±** 851592	.80655 **±** .578292	
**CXCL9(pg/ml)**	**Pretreatment**	.20700 **±** .102838	.19700 **±** .078764	.19854 **±** .134327	.18836 **±** .059980	0.492^a^, 0.555^b^, 0.256^c^, 0.239^d^, 0.174^e^, 0.393^f^
	**12 Weeks**	.15520 **±** .105935	.14029 **±** .039694	.28985 **±** .489042	.12573 **±** .053301	
**CXCL11(pg/ml)**	**Pretreatment**	.13520 **±** .080444	.13905 **±** .074375	.17885 **±** .093798	.16373 **±** .103128	0.144^a^, 0.190^b^, 0.787^c^, 0.435^d^, 0.065^e^, 0.634^f^
	**12 Weeks**	.15260 **±** .081185	.16190 **±** .098519	.17154 **±** .049891	.19991 **±** .152004	
**CXCL12(pg/ml)**	**Pretreatment**	51.01730 **±** 206.396544	.39581 **±** .154332	.58746 **±** .244147	.43218 **±** .163781	0.187^a^, 0.185^b^, 0.182^c^, 0.229^d^, 0.065^e^, 0.188^f^
	**12 Weeks**	.50380 **± .**065023	.42181 **±** .147961	.61662 **± .**196068	.42227 **± .**205439	
**CXCL13(pg/ml)**	**Pretreatment**	.35510 **±** .069974	.31505 **±** .105584	.45677 **±** .274538	.29830 **± .**105315	0.001^a^, 0.753^b^, 0.017^c^, 0.329^d^, 0.251^e^, 0.072^f^
	**12 Weeks**	.49510 **±** .139194	.42638 **±** .246432	.56569 **± .**271026	.33490 **±** .145816	
**CXCL16(ng/ml)**	**Pretreatment**	1.00250 **±** .101567	.87652 **± .**244527	1.16792 **± .**424545	1.01791 **±** .331579	0.000^a^, 0.081^b^, 0.227^c^, 0.689^d^, 0.378^e^, 0.702^f^
	**12 Weeks**	1.12440 **±** .298719	1.11633 **± .**323698	1.36885 **± .**342009	1.23936 **±** .417217	
**E-cadherin(ng/ml)**	**Pretreatment**	.82420 **±** .425530	.47852 **±** .436687	.98600 **±** .843119	.63418 **±** .660015	0.240^a^, 0.426^b^, 0.041^c^, 0.864^d^, 0.748^e^, 0.794^f^
	**12 Weeks**	.95180 **±** .666040	.55862 **±** .577851	1.09185 **±** .782186	.68536 **± .**657444	

*p-value comparing the effect of time*^a^*, treatment type*^b^*, response to treatment*^c^*, time-type interaction*^d^*, time-response interaction*^e^*, type-response interaction*^f^.

The same findings were observed in patients receiving pegylated interferon α2 a therapy, since CXCL5 pretreatment levels (1.220 ± 1.022 pg/ml) and (1.423 ± .925 pg/ml) were significantly higher than those assessed either after 12 (1.004 ± .699 pg/ml) and (0.808 ± 0.609 pg/ml) or 24 weeks (0.766 ± 0.433 pg/ml) and (0.862 ± 0.634 pg/ml) of non-responders and responders; respectively (p-value = 0.0001). No significant difference was found in relation to treatment type, response to treatment, time-type interaction, time-response interaction, or type-response interaction.

**Figure 2:**
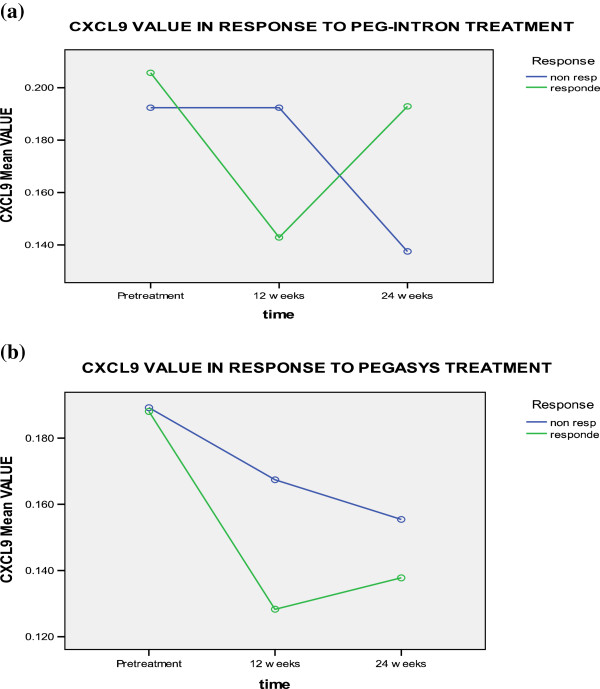
CXCL-9 levels in responders and non responders to a) PEG-Interferone and, b) pegasys at different time intervals.

CXCL9 levels decreased in responders to pegylated interferon α2b and α2a therapy after 12 and 24 weeks. Whereas in non-responders, CXCL9 levels decreased at 12 weeks of therapy, then it increased at 24 weeks. No significant difference was found for the effect of time, treatment type, response to treatment, time-type interaction, time-response interaction, or type-response interaction (Table 3 and Figure 2).

**Figure 3:**
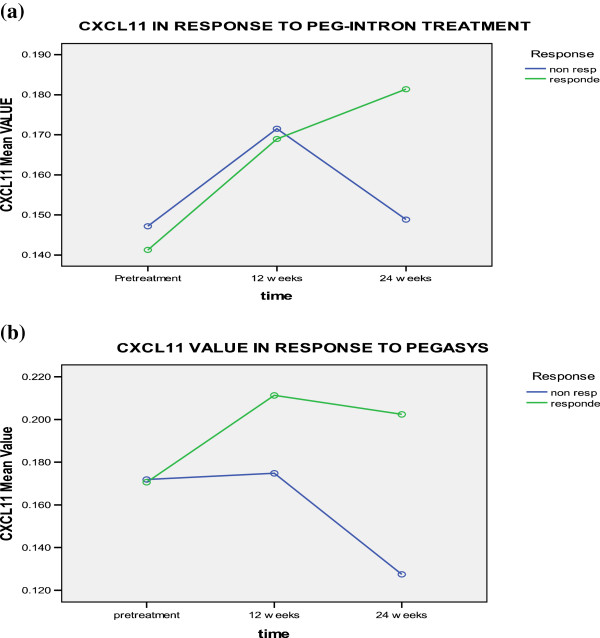
CXCL-11 levels in responders and non responders to a) PEG-Interferone and, b) pegasys at different time intervals.

Serum levels of CXCL11 were elevated in responders to pegylated interferon α2b therapy at 12 and 24 weeks, while it diminished at 12 weeks then increased at 24 weeks for both responders and non-responders to pegylated interferon α2a therapy. However, the difference was not statistically significant in relation to the effect of time, treatment type, response to treatment, time-type interaction, time-response interaction, or type-response interaction (Table 4 and Figure 3).

**Figure 4:**
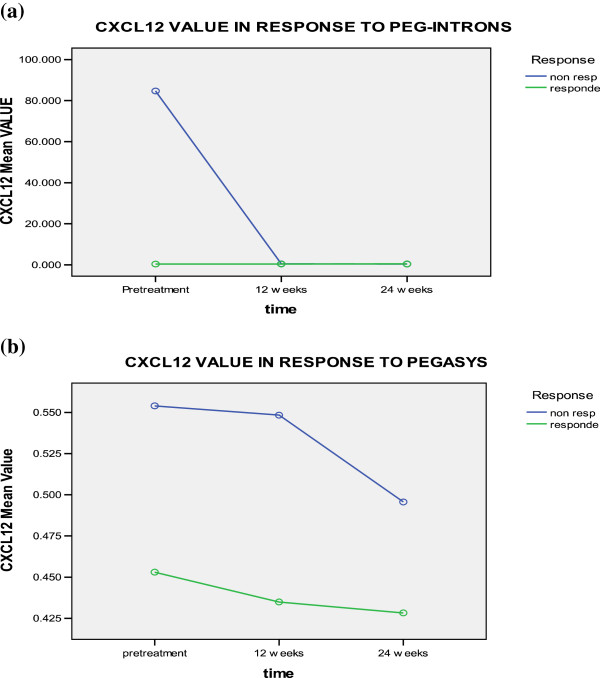
CXCL-12 levels in responders and non responders to a) PEG-Interferone and, b) pegasys at different time intervals.

CXCL12 levels showed slight increase in responders to peg-intron therapy at 12 weeks and decreased in non-responders. In patients receiving pegasys therapy, it decreased after 12 and 24 weeks, but no significant difference was found for time change, treatment type, response to treatment, time-type interaction, time-response interaction, or type-response interaction (Table 3 and Figure 4).

**Figure 5:**
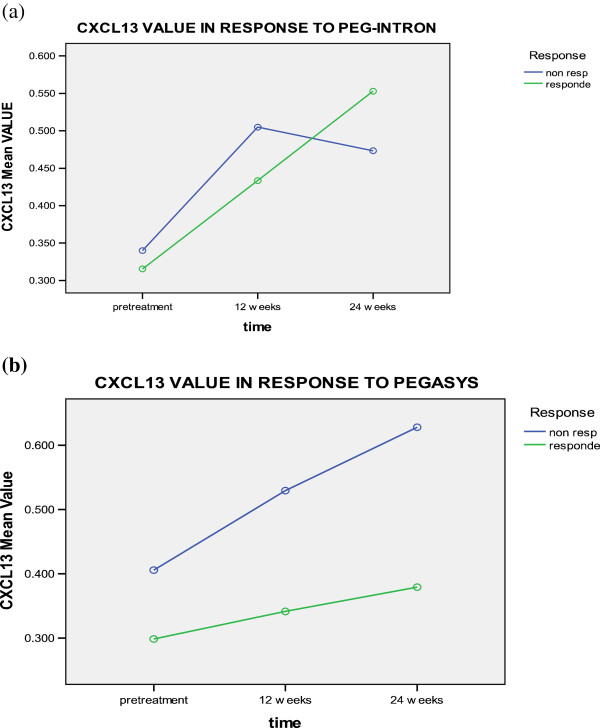
CXCL-13 levels in responders and non responders to a) PEG-Interferone and, b) pegasys at different time intervals.

Serum pretreatment levels of CXCL13 (0.405 ± 0.274 pg/ml and 0.298 ± 0.105 pg/ml) were significantly lower than those evaluated either at 12 (0.529 ± 0.271 pg/ml and 0.341 ± 0.145 pg/ml) or 24 weeks (0.628 ± 0.455 pg/ml and 0.399 ± 0.128 pg/ml) in pegylated interferon α2a treated patients for the non-responders and responders; respectively (p = 0.002). Similarly, CXCL13 pretreatment levels for responders of pegylated interferon α2b therapy (0.315 ±0.105 pg/ml) were significantly lower than those assessed either after 12 (0.433 ± 0.246 pg/ml) or 24 weeks (0.539 ±0.377 pg/ml). For the pegylated interferon α2a nonresponders, CXCL13 levels increased at 12 weeks from 0.340 ± 0.069 to 0.504 ± 0.139 pg/ml then, it decreased at 24 weeks to 0.473 ± 0.181 pg/ml (Table 3 and Figure 5). However, no significant difference was found in relation to treatment type, response to treatment, time type interaction, time-response interaction, or type-response interaction.

**Figure 6:**
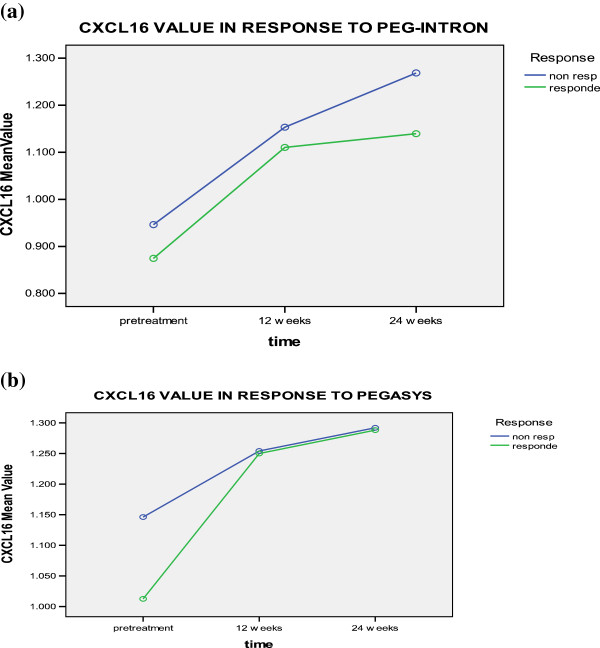
CXCL-16 levels in responders and non responders to a) PEG-Interferone and, b) pegasys at different time intervals.

Pretreatment levels of CXCL16 for non-responders to pegylated interferon α2b and α2a therapy; respectively (0.946 ± 0.101 and 1.146 ± 0.424 ng/ml) and responders (0.874 ± 0.244 & 1.012 ± 0.331 ng/ml) were significantly lower than those after 12 weeks for non-responders (1.153 ± 0.298 & 1.254 ± 0.342 ng/ml), and responders (1.110 ± 0.323 & 1.249 ± 0.417 ng/ml), or after 24 weeks from the beginning of therapy for non-responders (1.268 ± 0.353 & 1.291 ± 0.354 ng/ml) and responders (1.139 ± 0.408 & 1.288 ± 0.402 ng/ml) (Table 3 and Figure 6). However, no significant difference was found in relation to treatment type, response to treatment, time-type interaction, time-response interaction, or type-response interaction.

**Figure 7:**
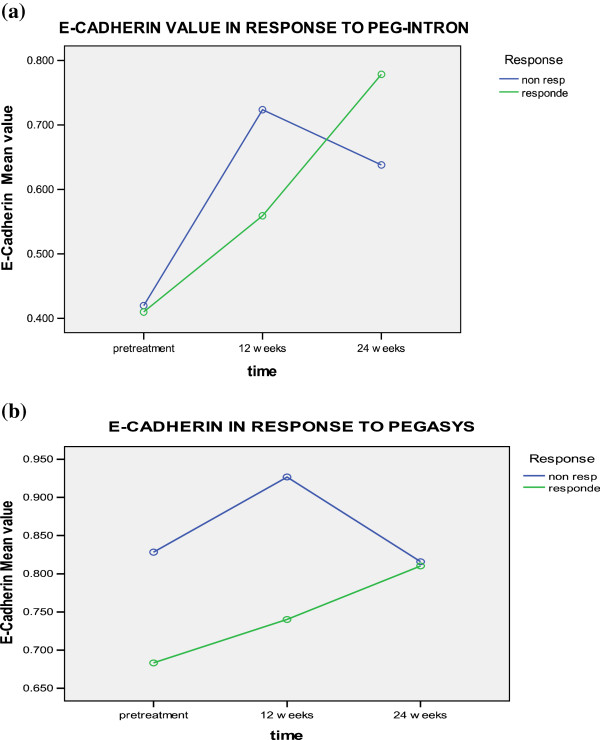
E-cadherin levels in responders and non responders to a) PEG-Interferone and, b) pegasys at different time intervals.

Serum levels of E-Cadherin increased observably in non-responders and responders after 12 weeks from the beginning of therapy (either Pegylated IFNα 2a or 2b groups) and continued to increase at 24 weeks but in responders only. No significant difference was found in relation to time change, treatment type, response to treatment, time-type interaction, timeresponse interaction, or type-response interaction (Table 3 and Figure 7).

No significant correlation was found between the expression levels of any of the studied chemokines and baseline HCV RNA in our studied patients.

## Correlations between different chemokines

Before treatment initiation CXCL13 was significantly correlated to CXCL9 (r = 0.385, p = 0.003), CXCL16 (r = 0.366, p = 0.048) and E-cadherin (r = 0.511, p = 0.000). While E-cadherin was significantly correlated to CXCL16 (r = 0.628, p = 0.000). Otherwise there was no significant correlation between the others chemokine’s studied or between them and the baseline AST, ALT and albumin levels except for the significant correlation between Ecadherin and ALT level (r = 0.363, p = 0.048).

At 12 weeks of therapy E-cadherin was significantly correlated to CXCL5 (r = 0.358, p = 0.047), CXCL9 (r = 0.311, p = 0.021), CXCL12 (r = 0.789, p = 0.000), CXCL13 (r = 0.583, p = 0.000), CXCL16 (r = 0.810, p = 0.000) and ALT levels (r = 0.311, p = 0.021).

CXCL5 was significantly correlated to CXCL9 (r = 0.359, p = 0.007), CXCL11(r = 0.456, p = 0.000), CXCL12 (r = 0.417, p = 0.002), CXCL13 (r = 0.448, p = 0.001) and CXCL16 (r = 0.326, p = 0.015). CXCL9 was significantly correlated to CXCL12 (r = 0.640, p = 0.000), CXCL13 (r = 0.422, p = 0.001) and CXCL16 (r = 0.519, p = 0.000). CXCL12 was significantly correlated to CXCL13 (r = 0.622, p = 0.000) and CXCL16 (r = 0.827, p = 0.000). CXCL13 was significantly correlated to CXCL16 (r = 0.531, p = 0.000).

At 24 weeks of therapy CXCL5 was significantly correlated to CXCL9 (r = 0.327, p = 0.027), CXCL11 (r = 0.587, p = 0.000), CXCL13 (r = 0.322, p = 0.029). CXCL13 was significantly correlated to CXCL9 (r = 0.311, p = 0.036), CXCL12 (r = 0.494, p = 0.000), CXCL16 (r = 0.400, p = 0.006) and E-cadherin (r = 0.454, p = 0.002) while CXCL12 was significantly correlated to CXCL16 (r = 0.634, p = 0.000) and E-cadherin (r = 0.777, p = 0.000). CXCL16 was significantly correlated to E-cadherin (r = 0.861, p = 0.000). Both CXCL16 and E-cadherin were significantly correlated to ALT levels (r = 0.319, p = 0.031) and (r = 0.350, p = 0.017); respectively.

## Discussion

Previous studies have investigated pretreatment predictors of response to interferon based therapy in HCV-associated CHC patients. Some of these studies have shown that cytokines could be used as predictors of treatment outcome [17].

In the current study, different baseline data that might affect the achievement of SVR and different chemokines levels were analyzed at different time intervals from starting therapy. We were able to show a statistically significant difference between responders and nonresponders to peg-intron and pegasys therapy in relation to AFP and AST values. Assessment of the correlation between the expression levels of these chemokine’s and response to treatment at baseline, week 12, and 24 revealed that the baseline serum level of CXCL5 was significantly higher than those after 12 or 24 weeks from the beginning of pegylated interferon α2a and α2b therapy for responders and non-responders but with no significant correlation with response to treatment. This could be attributed to an interaction between immune response and viral activity, which is indicated by the differences in cytokines and chemokine’s expression during the course of treatment [20]. On the other hand, Dou et al., [21] demonstrated that the HCV-2a core protein was shown to down-regulated CXCL5 gene in human hepatoma (Huh-7) cell line.

Our results regarding the decrease in the CXCL9 level in responders to pegylated interferon α 2 b and α 2 a therapy after 12 and 24 weeks are consistent with those of Wan et al., [20] who demonstrated that serum CXCL9 levels decreased significantly after the initiation of IFN therapy indicating a possible effect on the rate of viral clearance. On the other hand Butera et al., [22] found that pretreatment samples showed no consistent differences in CXCL9 level between patients who subsequently failed therapy and those who subsequently developed asustained response to antiviral therapy. They also found that CXCL9 level declined during therapy in both responders and non-responders. Among sustained responders, CXCL9 level remained low after completion of therapy while in non-responders, the drop in CXCL9 was transient. A possible explanation for the difference between our results and the later study could be our smaller sample size and the fact that all our patients were genotype 4.

Our data regarding the elevated CXCL11 level in responders of peg-intron therapy at 12 and 24 weeks, followed by a decrease at 12 weeks then an increase at 24 weeks for both responders and non-responders of pegasys therapy with no significant difference regarding time effect, treatment type, and response to treatment contrast with those of Butera et al., [22] who found that CXCL11 level measurement was lower in those who subsequently achieved SVR than in those who subsequently have no reduction in HCV RNA during or after antiviral therapy but with no significant differences in response. Therefore, they concluded that there was no association between pretreatment levels of CXCL11 and the outcome of treatment. The increase in CXCL11 level in the current study may be related to an increase in its receptor (CXCR3) levels in CHC patients as previously mentioned by Perney et al., [23]. We also reported a slightly increase in CXCL12 level was responders to PEG-IFN α2b therapy at 12 weeks and a decrease in non-responders and in all patients receiving PEG-IFN α2a after 12 and 24 weeks. Cicinnati et al., [24] have previously mentioned that IFN-α can modulate the expression of G protein coupled chemokine receptors, and some adhesion molecules, which are required for dendritic cell (DC) trafficking into secondary lymphoid tissue. Accordingly, the effect of IFN on CXCL12 levels, in the current study, could be explained by IFN-α therapy- induced migration of DC from inflamed hepatic portal areas towards secondary lymphoid tissue with increased chemotaxis to CXCL11 and CXCL12. Our results regarding the significant reduction of pretreatment CXCL13 level was with time of interferon treatment for both responders and non-responders regardless of treatment type are consistent with Sansonno et al. [25] who mentioned that up-regulation of CXCL13 gene expression is a distinctive feature of HCV-infected patients. Therefore high levels of this chemokine could be detected in the liver and skin of patients with CHC and these levels might decrease after therapy.

In our patients, pretreatment CXCL16 level increased significantly with time in responders and non-responders to pegylated interferon α2b and α2a therapy with no significant difference between the two groups. This could be attributed to the effect of IFN, which improves TH-1 response [26]. IFN-α treatment has been shown to regulate the activity of other cytokines and chemokines either directly or indirectly [27].

Alteration in E-Cadherin expression has been associated with human carcinogenesis including HCC [28]. In our series, E-Cadherin increased in non-responders and responders to Pegylated IFNα 2a or 2b after 12 weeks from the beginning of therapy however it continued to increase at 24 weeks only in responders of both groups. This could be attributed to the effect of HCV core protein, which represses E-Cadherin expression at the transcriptional level by down-regulating its promoter activity through promoter hypermethylation [29]. The significant difference in serum levels of E-Cadherin, reported in our study, at week 12 between responders and non-responders shows that it could be used as a predictor of EVR in IFN- treated CHC patients.

No significant correlation was found in the current study between any of the studied chemokines levels and baseline HCV RNA. On the other hand, baseline data have shown significant correlations between CXCL9 and CXCL13, CXCL13 and CXCL16, CXCL13 and E-cadherin, CXCL16 and E-cadherin, as well as between ALT level and E-cadherin that were maintained in follow up results.

In other words, these significant correlations were present at pretreatment, after 12 weeks, and after 24 weeks. In a previous study done by Butera et al., [22] stated that there was no correlations between CXCL9 and CXCL11 chemokines levels and viral load. On the other hand, Perney et al., [23] found a correlation between the expression of CXCR3 on peripheral blood CD4+ T cells and AST serum levels. Cicinnati et al., [24] also found that there were no significant correlations between systemic levels of CXCL12 and gender, age, grade of hepatic inflammation, stage of fibrosis, transaminases, bilirubin, HCV genotype or viral load. Also, Sansonno et al., [25] found that there was no correlation between CXCL13 concentrations and circulating viral load or ALT levels. One of the explanations for these findings is the lack of a direct effect of the virus on the sources of CXCL13 production.

## Conclusion

In conclusion, our results have shown that serum levels of CXCL13 and E-Cadherin have the potential to be used as serological markers at week 12 to the response of combined PEG IFN- α/RBV therapy. Also, significant changes with time have been found in the serum levels of CXCL5, CXCL13, and CXCL16 before pegylated interferon α 2 a and α 2 b therapy, and at weeks 12 and 24 with no significant difference in relation to treatment type, response to treatment, time-type interaction, time-response interaction, or type-response interaction. However, there were some limitations of our study, as example, this study should be done on a large scale in order to be validated. Also, it is better to apply one type of PEG-IFN preparations, and avoiding confounding was not an easy task, when assessing the association between tested chemokines and an outcome variable which leads to an overestimate or underestimate of the true association between exposure and outcome.

## Methods

### Patients

This prospective study included 57 patients with chronic HCV (CHC) liver disease. These patients constituted a part of a randomized controlled study including 200 patients receiving either pegylated interferon α 2b or pegylated interferon α 2a in combination with ribavirin during the period from September 2006 to March 2009. A written informed consent for immunological research, in which patients were assigned to give 10 mL of venous blood at each studied point during the course of therapy, blood was obtained from all patients before enrollment in the study. Only 57 patients accepted to share in the current study (32 received pegylated interferon α 2b and 25 received pegylated interferon α 2a, at baseline and after 12 and 24 weeks). All patients were treated in Al Qahira Al Fatimia Hospital (a district hospital at the middle of Cairo) as a part of the national program for combating viral hepatitis under supervision and sponsorship of The Ministry of Health. A written consent was obtained from all patients prior to enrollment in the study and the ethical committee of Al Qahira Al Fatimia Hospital and Ministry of Health approved the protocol, which was in accordance with the ethical guidelines of the Helsinki Declaration.

Selection criteria included male or female patients with CHC, aged 18 years or older with HBsAg negative, anti-nuclear antibody (ANA) <1:160, positive anti-HCV antibodies and HCV RNA by RT-PCR. All patients had white blood cells (WBCs) > 4000/mm3, neutrophil count >2000/mm3, platelets >75 000/mm3, prothrombin time <2 seconds above the upper limit of normal (ULN), direct bilirubin 0.3 mg/dL or within 20% of ULN, albumin >3.5, alpha fetoprotein <100, serum creatinine within normal limit (WNL), fasting blood sugar 115 mg/dl or within 20% ULN and if diabetic Hb A1C < 8.5% with normal T3, T4 and TSH. On the other hand, patients with decompensated liver disease or those with chronic liver disease due to causes other than HCV e.g. HBV, alpha-1 antitrypsin deficiency, Wilson’s disease, haemochromatosis, alcoholic liver disease or autoimmune disease were excluded from the study. Similarly, patients with hypersensitivity to interferon or ribavirin, pregnant or breast feeding females and those with any co-morbid conditions were excluded. All patients were subjected to full history taking and clinical assessment, routine laboratory work up including complete blood picture, liver biochemical profile, serum urea and creatinine, blood glucose, pregnancy test for married females, HBsAg, ANA, TSH, AFP and anti-schistosomal Ab titer. ECG was done for males > 40 years and females >50 years old. Ocular examination and abdominal ultrasound were also done.

Follow up and monitoring response to antiviral therapy was done on week 1, 2 and 4 after initiation of therapy then every 4 weeks. During each follow up, signs and symptoms of possible adverse effects due to the drugs were evaluated by routine laboratory studies. If treatment was continued in the presence of adverse effects, either dose adjustments were considered or the particular side effect was treated or monitored without lowering dosages depending on its severity.

Quantitative HCV RNA by Real time-polymerase chain reaction (RT-PCR) was performed after 12 weeks of therapy to determine Early Virological Response (EVR) upon which the decision to continue treatment after 12 weeks was taken, in addition to the patients’ tolerance to drugs and laboratory profile. Qualitative HCV viral load was also done after 24 weeks of treatment and if negative, the patient continued on the treatment. It was also done once the course of treatment was completed to document the End of Treatment Response (ETR). Sustained Virological Response (SVR) was defined as the absence of detectable HCV RNA in serum at the 24th week after the end of treatment [30].

## Quantitative real time PCR for determination of HCV viremia

RNA was extracted from patients’ blood using the QIAmp Viral RNA Mini Kit (QIAGEN, Santa Clarita, U.S.A) according to the manufacturer’s instructions, then quantitative Real time-polymerase chain reaction (RT-PCR) was performed using previously standardized realtime RT-PCR protocol for HCV, supplied from applied biosystems (USA) and according to Zekri et al. [31].

## HCV genotyping

HCV genotyping was done using the INNOLIPA–II technique as previously described by Zekri et al. [32].

## Monitoring patients’ response to therapy

Responses to therapy in patients with HCV were determined according to the consensus guidelines of the National Institutes of Health. Responders to therapy were defined by normalization of serum alanine aminotransferase (ALT) and absence of detectable serum HCV RNA at the end of treatment (48 weeks). Relapsed responders to therapy were defined by normalization of serum ALT and absence of detectable serum HCV RNA at the end of treatment but with an increase of the serum ALT and the presence of HCV RNA at follow-up (72 weeks). Non-responders were defined by elevated serum ALT and the presence of HCV RNA at the end of treatment.

## Assay of chemokines

Ten ml of blood were obtained from patients for serum separation before the start of therapy, after 12 and 24 weeks of therapy. CXCL5, CXCL9, CXCL11, CXCL12, CXCL 13, CXCL 16 chemokines and E-Cadherin were assayed using the quantitative ELISA plate method. The kits used were provided by Quantikine kits (R&D Systems, Inc. 614 McKinley Place NE Minneapolis, MN 55413 United States of America), sponsored by NCI and used according to the manufacturer’s instructions.

## Histopathological examination

Pretreatment liver biopsies from all patients were examined to confirm the diagnosis and determine the stage of fibrosis and the grade of inflammation using Modified Knodell score [33], in the form of 18 points for histopathology activity index (HAI), six points for stage of fibrosis and four points for stage of steatosis [34].

The histological activity index in the studied patients were graded into minimal (0–3), mild (4–8), moderate (9–12) and severe (13–18) points. The stage of fibrosis was graded into minimal (0–1), moderate (2–4) and marked (5–6) points.

## Statistical methods

Statistical analysis was done using SPSS version 17 statistical software package for windows. Quantitative variables were expressed by mean and SD (Standard deviation), comparison of means using Student’s *t*-test, Mann–Whitney *U*-test or ANOVA test were done when appropriate. Time-type interaction, time-response interaction, or type-response interaction were measured and analyzed statistically by repeated measures analysis of variance. In the table of multivariate tests, we took into consideration the p-values (for Wilks Lamda), and partial Eta square (according to Cohen’s standard) for measuring the interactions and their effect size. MANOVA was used under the same circumstances as ANOVA but when there were multiple dependent variables as well as independent variables within the model which the researcher wishes to test. MANOVA is also considered a valid alternative to the repeated measures ANOVA when sphericity was violated. Also, Pearson correlation test was performed for correlating quantitative variables. Qualitative variables were expressed by frequency and percent, comparison of mean values and assessment of relations using chisquared test or Fisher’s exact test was done when appropriate. P-value was considered significant when < 0.05 significant and highly significant when < 0.01.

## Abbreviations

ALT: Serum alanine aminotransferase; ANA: Anti-nuclear antibody; AFP: Alpha-fetoprotein; AST: Aspartate aminotransferase; CHC: Chronic hepatitis C; DC: Dendritic cells; EVR: Early virological response; ETR: End of treatment response; HAI: Histopathology activity index; HCC: Hepatocellular carcinoma; HCV: Hepatitis C virus; HBsAg: Hepatitis B surface antigen; PEG IFN-α/RBV: Pegylated interferon-alpha and ribavirin; SD: Standard deviation; SVR: Sustained virological response; TSH: Thyroid stimulating hormone; ULN: Upper limit of normal; WBCs: White blood cells; WHO: World health organization; WNL: Within normal limit.

## Competing interests

The authors declare that they have no competing interests.

## Authors’ contributions

ARNZ generated the idea, helped in revision of the manuscript, was responsible for the whole practical part, AAB managed the pathology work, helped in revision of the manuscript, WSM was help in editing, revising and did the statistical part of the manuscript, HMAED carried out the ELISA for the specified chemokines, shared in editing and revising the manuscript, HS was responsible for patient’s treatment and sampling wrote the draft of the manuscript, NZ managed the patients and their clinical part, DHE was help in editing, revising the manuscript and AO managed the patients and their clinical part, shared in the idea generation. All authors read and approved the final manuscript.

## Financial competing interests

The contents are solely the responsibility of the authors and do not necessarily represent the views of the funding source. The authors declare that they have no competing interests.
